# Effectiveness of Laser Acupuncture in Alleviating Chronic Insomnia: A Single-Blinded Randomized Controlled Trial

**DOI:** 10.1155/2019/8136967

**Published:** 2019-06-13

**Authors:** Chih-Kuang Chen, Yin-Chou Lin, Ju-Wen Cheng, Yu-Cheng Pei, Geng-Hao Liu, Yen-Lung Chen, Alice May-Kuen Wong

**Affiliations:** ^1^Department of Physical Medicine and Rehabilitation, Chang Gung Memorial Hospital, Taoyuan 33378, Taiwan; ^2^School of Medicine, Chang Gung University, Taoyuan 33302, Taiwan; ^3^Healthy Aging Research Center, Chang Gung University, Taoyuan 33302, Taiwan; ^4^Division of Acupuncture and Moxibustion, Department of Traditional Chinese Medicine, Chang Gung Memorial Hospital, Linkou 33305, Taiwan; ^5^Sleep Center, Chang Gung Memorial Hospital, Taoyuan 33378, Taiwan; ^6^School of Traditional Chinese Medicine, Chang Gung University, Taoyuan 33302, Taiwan; ^7^Graduate Institute of Clinical Medical Sciences, College of Medicine, Chang Gung University, Taoyuan 33302, Taiwan

## Abstract

**Study Objectives:**

This study investigates the therapeutic effect of Low Level Laser Therapy (LLLT) acupuncture for chronic insomnia.

**Methods:**

Thirty-seven adult subjects with chronic insomnia were recruited and randomly assigned to three groups, namely, Group A (6 pairs of acupoints: Ex-HN 22, HT 7, SP 6, KI 3, LR 3, and PC 6, bilaterally distributed); Group B (acupoints as for Group A other than PC 6, which was applied only on the left side [i.e., the dominant side of the PC meridian] and the addition of DU 20, which is the main tonic acupoint for integration of all the meridians); and a control group (sham LLLT). The subjects in the treatment groups (i.e., Groups A and B) received LLLT acupuncture and those in the control group received sham LLLT for 15 minutes per session twice a week for five weeks. The sleep quality of all the subjects was evaluated using the Pittsburgh Sleep Quality Index (PSQI), the Epworth Sleepiness Scale (ESS), the Hospital Anxiety and Depression Scale (HADS), and a sleep diary. In addition, the sympathetic activity before and after every treatment sessions was measured using the Heart Rate Variability (HRV).

**Results:**

All three groups showed an improved PSQI score. However, only Group A showed a significant reduction in the sleep onset latency and number of awakenings at night and a higher sleep efficiency and ESS score. Furthermore, Group B showed an increased low frequency power and normalized low frequency of the HRV signal and a lower normalized high frequency power, suggesting an increased sympathetic activity and decreased parasympathetic activity.

**Conclusions:**

For chronic insomnia insomniacs, LLLT appears to shorten the sleep latency, decrease the number of awakening events at night, and improve the sleep efficiency.

## 1. Introduction

Sleep plays a vital role in allowing the body to recover from fatigue and to repair [1, 2]. In addition, adequate sleep is essential to facilitate a reorganization of the body and mind following daily activities [3]. Long-term sleep deprivation, or a decreased quality of sleep, is associated with many physical and mental illnesses, including impaired immunity, endocrine disease, mood swings, an inability to pay attention, reduced work performance, and a higher risk of accidents [4].

A recent survey showed that around 20% of the adult population have some form of sleep complaint [5]. As a result, sleep disturbance has extremely important ramifications for public health. Sleep disorders are usually treated by pharmacotherapy. However, this may cause undesirable, and indeed dangerous, side effects such as transient memory loss, daytime drowsiness, poor balance, and gait difficulties [6, 7]. To this end, 65% of patients with sleep disorders have negative views on the use of hypnotics and 67% of them are willing to try nonpharmacological therapy [5].

Common nonpharmacological interventions for insomnia include acupuncture, Cognitive Behavioral Therapy (CBT), neurofeedback therapy, bright light therapy, music therapy, or some form of complementary and alternative medicine (CAM) therapy [8]. Acupuncture has been used in China for centuries [9] to control the stress, anxiety, depression, medical, and physical problems induced by insomnia [10–14]. However, needle acupuncture can elicit a variety of unpleasant side effects, including pain and local hematoma. Thus, its acceptance among patients tends to be limited. The use of noninvasive laser acupuncture has therefore attracted growing interest over the past decades. Laser acupuncture can be traced back to the work of Mester in 1966, who developed a Low Level Laser Therapy (LLLT) biostimulation technique with benefits analogous to those of needle acupuncture, but with the advantages of noninvasiveness and an absence of side effects [15]. LLLT has since been widely applied to many traditional acupuncture points [16, 17] where the needles traditionally used to apply biostimulation are replaced by electromagnetic waves [18].

This study performs a single-blinded randomized controlled trial to evaluate the effectiveness of laser acupuncture for patients with chronic insomnia. The investigation focuses particularly on the effects of LLLT on the quality of sleep, daytime drowsiness, and balance of the autonomic system. In performing the study, it is hypothesized that laser acupuncture improves the sleep quality of patients with chronic insomnia and its effects can be determined by the choice of acupoint ensemble.

## 2. Materials and Methods

### 2.1. Criterion

The inclusion criteria for the present study were as follows: (1) Adult patients manifesting symptoms of insomnia according to the criteria laid down in the International Classification of Sleep Disorders 3rd edition (ICSD-3) such as difficulty initiating or maintaining sleep or waking up too early for at least 3 nights per week, for at least 3 months [19]. (2) A Pittsburgh Sleep Quality Index (PSQI) score of 5 or more [20]. (3) At least one month hypnotic free and willing to be free of hypnotics during the entire experimental period. The exclusion criteria were stated as follows: (1) Patient Health Questionnaire-9 (PHQ-9) scores of 10 or more, indicating a depressive tendency [21]. (2) Pregnancy or lactation. (3) History of clinically significant head trauma (e.g., brain damage), chemotherapy-induced peripheral neuropathy (CIPN), seizure disorder, psychosis, depression, or mania. (4) Current mood disorder. (5) Drug addiction or alcoholism.

### 2.2. Assessment Tools

*(1) Pittsburgh Sleep Quality Index (PSQI). *The PSQI is a self-administered questionnaire that is widely used in clinical sleep quality and disability studies and differentiates “good” from “poor” sleep by measuring seven domains, namely subjective sleep quality, sleep latency, sleep duration, habitual sleep efficiency, sleep disturbances, use of sleep medication, and daytime dysfunction over the last month. Each question is scored from 0 to 3, with the individual scores being summed to yield a global score ranging from 0 to 21, where a higher score indicates a poorer quality of sleep. Poor sleep is generally characterized as a total PSQI score of 5 or more [22].

*(2) Epworth Sleepiness Scale (ESS).* The ESS scale assesses the rate and severity of drowsiness in eight scenarios, including sitting, reading, watching television, and taking a car. Each question is scored from 0 (least severe) to 3 (most severe), giving a total possible score of 24 [23, 24].

*(3) Hospital Anxiety and Depression Scale (HADS). *The HADS scale is a self-assessment scale for detecting states of depression and anxiety. Each domain, i.e., depression and anxiety, is measured using seven questions. Each question is scored from 0 (lowest degree) to 3 (highest degree), giving a total possible score of 21 in each domain [25].

*(4) Sleep Diary. *Sleep diaries are widely used for the “subjective” assessment of sleep and typically contain the self-reported daily bedtime, the sleep duration, the number of nightly awakenings, the wake-up time, the nap history, the alcohol/caffeine intake, the self-feeling at bedtime/noon/afternoon, and so on [26].

*(5) Heart Rate Variability (HRV) Analysis. *In the present study, a Heart Rate Variability (HRV) analyzer (WE-MD-ANSA-01, Wegene Technologies Inc., Taiwan) was used to record the electrocardiography signals of the patients through electrode patches placed on the bilateral forearms with a sampling frequency of 256 Hz. The signals were recorded for 5 minutes with the subjects resting in the supine position on a bed. An offline analysis was then performed to determine the power in the high and lower frequency domains of the HRV signal as indicators of the parasympathetic and sympathetic activities, respectively [27, 28].

### 2.3. Experimental Equipment

**Figure 1:**
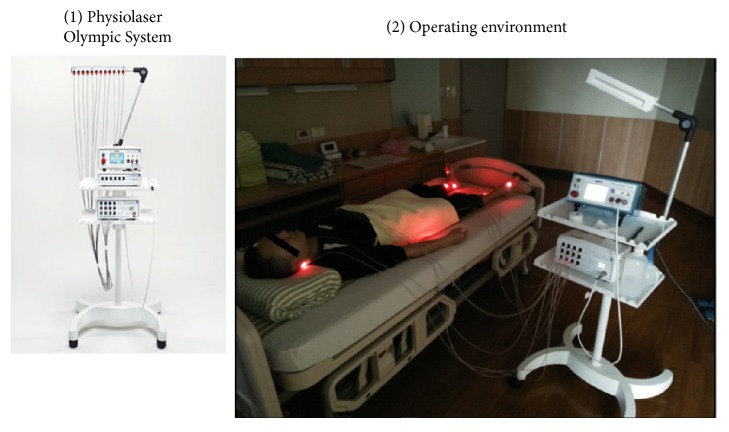
Experimental equipment and the operating environment.

LLLT acupuncture was delivered by a 12-beam Physiolaser Olympic system (RJ Laser, Winden, Germany) (see Figure 1).

### 2.4. Experimental Procedure

#### 2.4.1. Ethical Considerations

The experimental procedures were specifically approved by the Institutional Review Board (IRB) of Chang Gung Medical Foundation (Taiwan). Each subject provided informed consent prior to participation.

#### 2.4.2. Subjects

Subjects with chronic insomnia were recruited and their basic data, including gender, age, body weight, body height, and personal health details (Patient Health Questionnaire-9, PHQ-9), were collected. The assessment scales (PSQI, ESS, and HADS) and the use of the sleep diary were explained to the subjects in detail. The subjects were randomly allocated and assigned into three groups, namely, Group A (received laser acupuncture to the bilateral Anmian Ex-HN 22, Shenmen HT 7, Sanyinjiao SP 6, Taixi KI 3, Taichong LR 3, and Neiguan PC 6 acupoints); Group B (received laser acupuncture to the same acupoints as Group A other than Neiguan PC 6 which was applied only on the left side [i.e., the dominant side of the PC meridian] and Baihui DU 20 [i.e., the main tonic acupoint for integration of all the meridians [29]]); and a control group (received sham laser acupuncture to the same acupoints as Group A) (Table 1).

The subjects in Groups A and B received laser acupuncture to the 12 aforementioned acupoints, while those in the control group received sham laser treatment (using only the aiming beam). The subjects in the treatment groups (i.e., Groups A and B) received LLLT acupuncture and those in the control group received sham LLLT for 15 minutes per session twice a week for five weeks (10 sessions totally). The PSQI, ESS, HADS, and sleep diary were assessed in all the three groups before the first session and after the 10th session. Before and after each acupuncture session, the subjects received HRV assessment for 5 minutes.

#### 2.4.3. Statistical Analyses

Statistical analyses were performed using SPSS 17.0 software. The data were presented as mean ± standard deviation. The baseline characteristics data of the three groups were analyzed using ANOVA for the numerical data and Fisher's exact test for the categorical data such as level of education. Two-way ANOVA tests with repeated measures (groups *∗* times) were performed to compare differences. The dependent variables included the PSQI, ESS, and HADS scores, the sleep diary entries, and the HRV signal features. The level of statistical significance was set as P <0.05 in every case.

## 3. Results

**Figure 2:**
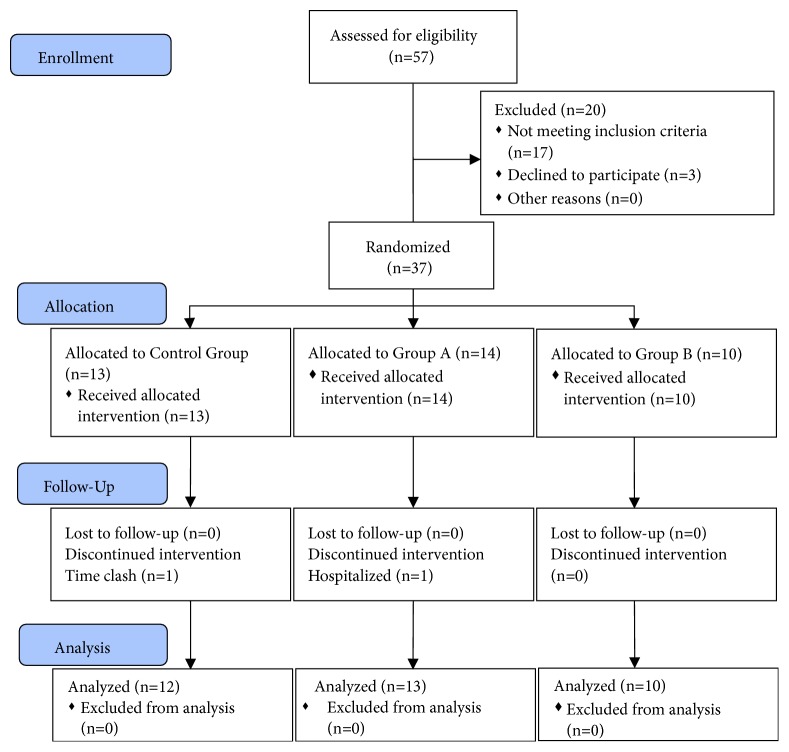
CONSORT flow diagram.

A total of 37 subjects participated in the study. Of these 37 subjects, 35 received full-course intervention, while 2 dropped out due to being too busy to attend the LLLT sessions and hospitalization for an unrelated issue, respectively (see Figure 2). No significant difference was found in the age, body weight, body height, personal health questionnaire scores, and level of education of the three groups (Table 2).

All three groups showed a significant improvement in the PSQI score following intervention (Table 3). No significant difference was found in the PSQI improvement of the three groups (Interaction effect P = 0.196). Regarding the ESS score, a significant postintervention improvement was found only for Group A (from 5.23±2.65 to 6.69±3.28, P < 0.05). By contrast, the ESS score reduced for both Group B and the control group. The HADS-anxiety score was significantly improved only in Group B (from 4.90±3.51 to 2.40±2.68, P < 0.01). LLLT intervention failed to significantly improve the HADS-Depression score in any of the three groups.

The sleep diary results (Table 4) showed that only Group A exhibited a significantly reduced sleep onset latency (from 45.00±31.62 to 25.00±26.62, P < 0.05) and number of awakenings at night (from 2.38±1.12 to 1.23±0.83, P < 0.01) or a better sleep efficiency (from 75.41±13.25% to 83.94±13.31%, P < 0.05). Regarding the HRV signal, only Group B showed a significant increase of VLFp, indicating an increase of sympathetic activity (from 6.21±0.88 to 6.72±0.61, P < 0.05) after the first intervention session (Table 5). No significant changes were observed in any of the other HRV parameters for any of the three groups (Table 5).

After the final intervention session, Group B was the only group to exhibit a significant change in the HRV parameters following LLLT treatment (Table 6). In particular, the group showed a significantly greater low frequency power (LFp) (from 4.58±1.05 to 5.55±1.15, P < 0.05) and normalized low frequency (LFnu) (from 32.21±13.51% to 51.03±17.15%, p=0.010, P < 0.05), implying a greater sympathetic activity. Group B additionally showed a significantly lower normalized high frequency (HFnu) (from 49.54±10.46% to 34.26±16.04%, P < 0.01), implying a decreased parasympathetic activity. Finally, Group B showed a significant reduction in the LF:HF ratio (from -0.48±0.62 to 0.44±0.82, P < 0.01), indicating a change in the balance of the autonomic activities.

## 4. Discussion

Acupuncture has been used as a traditional treatment for insomnia in China for centuries. Several randomized controlled clinical trials have confirmed the positive effects of acupuncture in treating insomnia [29, 30]. The effectiveness of acupuncture as a treatment mode is supported by many neuroendocrinological studies, which have shown that stimulation of certain acupoints modulates a wide variety of neuroendocrinological factors, including norepinephrine, melatonin, gamma-aminobutyric acid, and *β*-endorphin [14].

Laser acupuncture is regarded as safer than needle acupuncture and more appropriate for stimulating difficult-to-reach points, such as the acupoints located at the thoracic cage, including SP 20, KI 22 – 27, LV 13 and LV 14 which had higher risk for pneumothorax [31]. Furthermore, studies have shown that the effectiveness of laser acupuncture approaches that of traditional forms of acupuncture when treating myofascial pain, postoperative nausea/vomiting, and chronic tension headache [14]. The present study has evaluated the effectiveness of LLLT acupuncture in alleviating chronic insomnia using a single-blinded randomized controlled design and sham laser treatment as a control. The acupoints used in the LLLT sessions have been chosen in accordance with the fundamental Chinese medicine concept of Yin and Yang. Yang is related to Qi, i.e., warmth, light, activity, and daylight (the sun traveling through the heavens), while Yin is associated with blood, nourishment, darkness, passiveness, and night (the moon traveling through the night) [32]. The relationship between Yin and Yang is manifested in the waxing and waning of the day and night and is analogous to the periodicity of circadian rhythms within the human body. The inability to sleep at night is hence a manifestation of unwarranted Yang during the Yin phase or simply an insufficiency of Yin to counter Yang. This relationship of imbalance may be caused by Heart (HT 7) and Spleen (SP 6) deficiency, Heart (PC 6) and Kidney (KI 3) disharmony, hyperactivity of the Liver (Liv 3), or Heart Yin deficiency and Baihui (DU 20), causing imbalance of the body harmony and sleep disturbance. Insomnia may, however, also be related to the extraordinary Anmian acupoint (Ex-HN 22) [29].

All three groups showed a significant improvement in the PSQI score following LLLT intervention. This finding may be explained by the fact that all of the groups (including the control group) also received CBT as a basic intervention in this study. That is, the placebo effect of any kind of any intervention cannot be entirely ruled out [33]. CBT is designed to help the subject's self-perception or attitudes regarding insomnia [34]. Hence, Jacobs and his colleagues (2004) suggested that individuals suffering from insomnia should accept CBT as the first-line mode of treatment [35]. However, CBT is limited in the sense that it relies on the subject's willingness and persistency to make behavioral and lifestyle changes.

Significant improvements in the sleep onset latency, number of awakenings at night, and sleep efficiency were observed only in Group A. Likewise, a lower ESS score was also found only for Group A. By contrast, significant changes in the HRV signal after LLLT treatment were observed only in Group B, for which LLLT biostimulation was applied to an additional acupoint (DU 20). A significant reduction of anxiety was also observed only in Group B. The change of sympathetic and parasympathetic activities in Group B and the lowering of anxiety suggest that DU 20 (Baihui) is a powerful acupoint for this purpose.

In general, the results obtained in this study suggest that patients with difficulty falling asleep, or those who experience frequent awakenings at night, a lack of sufficient quality sleep, and episodes of daytime drowsiness, may find laser acupuncture of the Yin meridian helpful in alleviating these symptoms. If the patient additionally suffers anxiety and imbalance of the autonomic system, acupuncture of DU 20 may also be of benefit.

This study has several limitations, including a small sample size (n=37), a diversity of age and sex and a diversity of etiology of chronic insomnia including menopausal women (n=12), cancer patients in convalescence (n=3), and chronic stroke survivors (n=3). In particular, the standard protocol used in this study may not be appropriate for patients with such a wide variety of characteristics. It is possible that more personalized treatment modes may be more effective. Thus, further studies are required to determine the effectiveness of personalized laser acupuncture and to evaluate the long-term effects of such therapy.

## 5. Conclusions

The present results suggest that laser acupuncture may be an effective intervention for the relief of insomnia since it can shorten the sleep latency, reduce the number of awakenings at night, improve sleep efficiency, lower anxiety, and reduce daytime drowsiness. However, acupuncture of tonic acupoint DU 20 may be effective only in decreasing anxiety rather than alleviating insomnia.

## Figures and Tables

**Table 1:** Acupoints and therapy protocol in the three groups.

	Control Group	Group A	Group B
Acupoints			
Anmian (Ex-HN 22)	$\overset{ˇ}{\;}$	$\overset{ˇ}{\;}$	$\overset{ˇ}{\;}$
Shenmen (HT 7)	$\overset{ˇ}{\;}$	$\overset{ˇ}{\;}$	$\overset{ˇ}{\;}$
Neiguan (PC 6)	$\overset{ˇ}{\;}$	$\overset{ˇ}{\;}$	△
Sanyinjiao (SP 6)	$\overset{ˇ}{\;}$	$\overset{ˇ}{\;}$	$\overset{ˇ}{\;}$
Taixi (KI 3)	$\overset{ˇ}{\;}$	$\overset{ˇ}{\;}$	$\overset{ˇ}{\;}$
Taichong (LR 3)	$\overset{ˇ}{\;}$	$\overset{ˇ}{\;}$	$\overset{ˇ}{\;}$
Baihui (DU20)	—	—	○

Laser protocol	Sham laser	Nogier F-Continuous Beam 600mW 540J 15min	

*Note.* $\overset{ˇ}{\;}$: bilateral; △: only left side; ○: only add in Group B.

**Table 2:** Comparison of demographic data of three groups.

	Control Group (n=12)	Group A (n=13)	Group B (n=10)	*p*-value
Male / Female	2/10	3/10	0/10	0.280
Age (Year)	52.17±10.08	52.69±11.79	49.30±12.77	0.640
Body height (cm)	159.08±8.69	159.85±6.78	157.00±4.37	0.774
Body weight (kg)	62.83±14.31	60.19±8.65	56.40±7.40	0.409

PHQ-9	5.58±2.64	6.38±1.98	5.10±3.48	0.736
PSQI	12.42±3.00	11.69±3.20	13.20±3.88	0.534
ESS	8.67±5.79	5.23±2.65	7.00±5.42	0.270
HADS-Anxiety	6.92±4.34	5.54±3.33	4.90±3.51	0.394
HADS-Depression	4.50±3.34	3.69±2.18	3.40±3.24	0.674

Level of education				
Uneducated	0	0	0	0.158
Elementary school	0	0	0	
Junior high school	1	2	1	
Senior high school	7	5	1	
College	4	6	8	

*∗p*<0.05.

**Table 3:** Comparison of change in scores in questionnaire assessments among three groups.

	Baseline	After last intervention	Within-group *p*-value
PSQI			
Control Group	12.42±3.00	8.58±2.91	0.005*∗∗*
Group A	11.69±3.20	9.31±3.92	0.037*∗*
Group B	13.20±3.88	8.10±3.48	<0.001*∗∗∗*
ESS			
Control Group	8.67±5.79	7.83±3.95	0.490
Group A	5.23±2.65	6.69±3.28	0.028*∗*
Group B	7.00±5.42	5.00±3.59	0.148
HAD-Anxiety			
Control Group	6.92±4.34	4.75±2.96	0.086
Group A	5.54±3.33	5.00±2.86	0.536
Group B	4.90±3.51	2.40±2.68	0.007*∗∗*
HAD-Depression			
Control Group	4.50±3.34	2.83±0.80	0.107
Group A	3.69±2.18	3.31±2.81	0.445
Group B	3.40±3.24	1.90±2.64	0.062

*∗p*<0.05; *∗∗p*<0.01; *∗∗∗p*<0.001.

**Table 4:** Comparison of sleep diary among three groups.

	Baseline	After last intervention	Within-group *p*-value
TIB *(min.)*			
Control Group	466.67±74.63	434.58±79.10	0.385
Group A	457.31±101.71	440.77±81.88	0.643
Group B	423.00±89.45	452.00±104.67	0.280
SOL *(min.)*			
Control Group	57.58±64.57	38.33±30.48	0.338
Group A	45.00±31.62	25.00±26.62	0.013*∗*
Group B	37.00±17.03	43.80±49.83	0.725
NWAK *(times)*			
Control Group	2.25±1.36	1.67±1.72	0.253
Group A	2.38±1.12	1.23±0.83	0.002*∗∗*
Group B	1.80±1.40	1.50±1.27	0.279
TST *(min.)*			
Control Group	325.00±68.69	312.08±8.20	0.711
Group A	341.92±98.86	364.62±67.31	0.570
Group B	317.00±71.81	333.00±87.69	0.524
SE (%)			
Control Group	70.28±12.27	72.71±18.43	0.697
Group A	75.41±13.25	83.94±13.31	0.030*∗*
Group B	76.06±14.47	74.66±16.19	0.733

*∗p*<0.05; *∗∗p*<0.01; *∗∗∗p*<0.001.

*Note. *TIB: time in bed; SOL: sleep onset latency; NWAK: number of awakenings; TST: total sleep time; SE: sleep efficiency = (TST/TIB)*∗*100%.

**Table 5:** Change in HRV parameters after first intervention session.

	Before 1^st^ intervention	After 1^st^ intervention	Within-group *p*-value
Tp			
Control Group	6.34±0.94	6.62±1.05	0.240
Group A	6.78±0.90	6.73±0.78	0.798
Group B	7.05±0.73	7.32±0.55	0.080
VLFp			
Control Group	5.84±0.99	6.05±1.05	0.409
Group A	6.09±0.96	5.97±0.80	0.710
Group B	6.21±0.88	6.72±0.61	0.017*∗*
LFp			
Control Group	4.65±0.96	4.89±1.10	0.387
Group A	5.11±1.15	5.09±1.13	0.921
Group B	5.50±0.85	5.66±0.69	0.378
HFp			
Control Group	4.18±1.08	4.53±1.24	0.116
Group A	4.94±1.10	4.99±0.97	0.739
Group B	5.16±1.07	5.34±0.85	0.528
LFnu			
Control Group	51.66±15.13	49.56±16.60	0.668
Group A	45.75±14.96	44.85±17.99	0.821
Group B	50.03±20.08	49.26±8.85	0.909
HFnu			
Control Group	33.02±11.92	33.70±12.21	0.864
Group A	37.88±11.56	40.55±14.92	0.493
Group B	36.40±15.68	36.18±8.04	0.970
LF:HF			
Control Group	0.47±0.68	0.36±0.80	0.640
Group A	0.25±0.56	0.10±0.82	0.362
Group B	0.34±0.91	0.32±0.39	0.942

*∗p*<0.05; *∗∗p*<0.01; *∗∗∗p*<0.001.

**Table 6:** Change in HRV parameters after last intervention session.

	Before 10^th^ intervention	After 10^th^ intervention	Within-group *p*-value
Tp			
Control Group	6.16±1.01	6.38±1.07	0.311
Group A	6.83±0.79	7.04±0.62	0.111
Group B	6.53±1.03	7.23±1.47	0.346
VLFp			
Control Group	5.52±1.15	5.82±1.14	0.335
Group A	6.03±0.77	6.47±0.63	0.056
Group B	5.69±1.38	6.56±1.62	0.145
LFp			
Control Group	4.63±0.80	4.84±1.03	0.390
Group A	5.45±1.16	5.23±1.02	0.495
Group B	4.58±1.05	5.55±1.15	0.011*∗*
HFp			
Control Group	4.01±1.25	3.96±1.43	0.730
Group A	4.90±0.84	4.94±0.96	0.825
Group B	5.06±0.93	5.12±1.36	0.867
LFnu			
Control Group	54.57±14.16	58.93±14.21	0.403
Group A	54.52±15.92	48.01±19.01	0.123
Group B	32.21±13.51	51.03±17.15	0.010*∗*
HFnu			
Control Group	31.49±11.33	27.52±11.73	0.315
Group A	32.15±11.31	37.13±14.72	0.141
Group B	49.54±10.46	34.26±16.04	0.006*∗∗*
LF:HF			
Control Group	0.62±0.78	0.88±0.89	0.246
Group A	0.56±0.72	0.28±0.88	0.161
Group B	-0.48±0.62	0.44±0.82	0.007*∗∗*

*∗p*<0.05; *∗∗p*<0.01; *∗∗∗p*<0.001.

## Data Availability

The processed data required to reproduce these findings cannot be shared at this time as the data is concerning about the privacy of subjects.
